# MOS Capacitance Measurements for PEALD TiO_2_ Dielectric Films Grown under Different Conditions and the Impact of Al_2_O_3_ Partial-Monolayer Insertion

**DOI:** 10.3390/nano10020338

**Published:** 2020-02-17

**Authors:** William Chiappim, Marcos Watanabe, Vanessa Dias, Giorgio Testoni, Ricardo Rangel, Mariana Fraga, Homero Maciel, Sebastião dos Santos Filho, Rodrigo Pessoa

**Affiliations:** 1 i3N, Departamento de Física, Universidade de Aveiro, Campus Universitário de Santiago, 3810-193 Aveiro, Portugal; 2 Laboratório de Sistemas Integráveis, Escola Politécnica da Universidade de São Paulo (USP-SP), 05508-010 São Paulo, Brazil; marcosnwatanabe@gmail.com (M.W.); rrangel@lsi.usp.br (R.R.); sgsantos@lsi.usp.br (S.d.S.F.); 3 Laboratório de Plasmas e Processos (LPP), Instituto Tecnológico de Aeronáutica (ITA), 12228-900 São José dos Campos, Brazil; van_ametista@yahoo.com.br (V.D.); giorgiotestoni@gmail.com (G.T.); homero@ita.br (H.M.); 4 Instituto de Ciência e Tecnologia, Universidade Federal de São Paulo, 12231-280 São José dos Campos, Brazil; mafraga@ieee.org; 5 Instituto Científico e Tecnológico, Universidade Brasil, 08230-030 São Paulo, Brazil

**Keywords:** plasma-enhanced atomic layer deposition, titanium dioxide, nanolaminate, metal-oxide-semiconductor, electrical properties

## Abstract

In this paper, we report the plasma-enhanced atomic layer deposition (PEALD) of TiO_2_ and TiO_2_/Al_2_O_3_ nanolaminate films on p-Si(100) to fabricate metal-oxide-semiconductor (MOS) capacitors. In the PEALD process, we used titanium tetraisopropoxide (TTIP) as a titanium precursor, trimethyl aluminum (TMA) as an aluminum precursor and O_2_ plasma as an oxidant, keeping the process temperature at 250 °C. The effects of PEALD process parameters, such as RF power, substrate exposure mode (direct or remote plasma exposure) and Al_2_O_3_ partial-monolayer insertion (generating a nanolaminate structure) on the physical and chemical properties of the TiO_2_ films were investigated by Rutherford backscattering spectroscopy (RBS), Raman spectroscopy, grazing incidence X-ray diffraction (GIXRD), and field emission scanning electron microscopy (FESEM) techniques. The MOS capacitor structures were fabricated by evaporation of Al gates through mechanical mask on PEALD TiO_2_ thin film, followed by evaporation of an Al layer on the back side of the Si substrate. The capacitors were characterized by current density-voltage (J-V), capacitance-voltage (C-V) and conductance-voltage (G-V) measurements. Our results indicate that RF power and exposure mode promoted significant modifications on the characteristics of the PEALD TiO_2_ films, while the insertion of Al_2_O_3_ partial monolayers allows the synthesis of TiO_2_/Al_2_O_3_ nanolaminate with well-spaced crystalline TiO_2_ grains in an amorphous structure. The electrical characterization of the MOS structures evidenced a significant leakage current in the accumulation region in the PEALD TiO_2_ films, which could be reduced by the addition of partial-monolayers of Al_2_O_3_ in the bulk of TiO_2_ films or by reducing RF power.

## 1. Introduction

Titanium dioxide (TiO_2_) thin films and nanolaminates have a significant number of promising applications in different areas, such as microelectronics [1,2,3,4,5,6], photovoltaics [7,8], photocatalysis [9,10,11], fuel cells [12], sensors [13,14,15,16], anti-reflective coating applications [17], biomedical coatings [18,19] and food packaging applications [20]. The properties and applicability of TiO_2_ thin films are intrinsically related to their crystal structure. In this sense, they have been mainly produced in amorphous, anatase and/or rutile structure forms by a wide range of techniques, namely sol-gel [21,22], magnetron sputtering [23,24], chemical vapor deposition (CVD) [25], physical vapor deposition (PVD) [26], atomic layer deposition (ALD) [27,28] and plasma-enhanced atomic layer deposition (PEALD) [29,30,31,32,33,34]. Among them, PEALD requires lower substrate and process temperatures to obtain crystalline films [35]. It is also one of the most promising technologies for the growth of conformal coatings and nanolaminates in various structures and topographies [32,36] with layer thickness precisely defined by self-limited surface reactions [37]. During the PEALD of metal oxide films, oxygen (O_2_) plasma is used as an oxidant source [38]. The plasma is composed of a mixture of electrons, ions, neutrals and excited molecules/atoms. The action of each specie depends on the plasma exposure mode that can be remote or direct, where the latter allows contact of all plasma species with surface ligand groups [39]. The oxidizing plasma source used in the PEALD process has the advantage of reducing the purge time after the precursor pulse. Furthermore, its high reactivity enhances surface chemical reactions, allowing the process to be carried out at lower temperature and, consequently, preventing the interlayer diffusion that is responsible for the degradation of nanoscale device properties [40].

Because of its dielectric properties, TiO_2_ thin films are widely used in microelectronic and photovoltaic applications. In microelectronic devices and systems, the metal oxide deposition temperatures need to be below 500 °C because capacitors are expected to be deposited after the formation of transistors [41]. The same caution is required for the insulator/oxide deposition temperature in photovoltaic cells since temperatures above 300 °C cause degradation in these devices related to one order vacancy compound (OVC) [42]. The PEALD technique fits the requirements for both aforementioned devices, as it offers excellent uniformity, conformability and atomic level control of the growing oxide layer [43]. Moreover, as previously stressed, TiO_2_ growth can be conducted at lower process temperatures and under atmospheres with shorter purge pulses in PEALD reactors than in standard thermal ALD [44].

In our previous study [45], the effects of O_2_ plasma power, titanium precursor chemistry and plasma exposure mode on the growth mechanism, structure and morphology of PEALD TiO_2_ films were investigated. Herein, we extend this study with the focus on the fabrication and characterization of TiO_2_/p-Si MOS capacitors for potential applications in microelectronic and photovoltaic devices. J-V, C-V and G-V characteristics of the MOS capacitors at room temperature were investigated in function of the following PEALD process parameters: (i) RF power, (ii) plasma exposure mode and (iii) Al_2_O_3_ partial monolayer insertion (generating a nanolaminate structure). In addition, we also discuss how the plasma exposure affects or causes surface modifications and changes in electrical parameters of TiO_2_ films. There is a lack of available literature on the electrical characteristics of MOS capacitors formed by PEALD TiO_2_-based films on p-type Si substrates grown under different conditions.

## 2. Materials and Methods

### 2.1. Film Preparation

**Figure 1:**
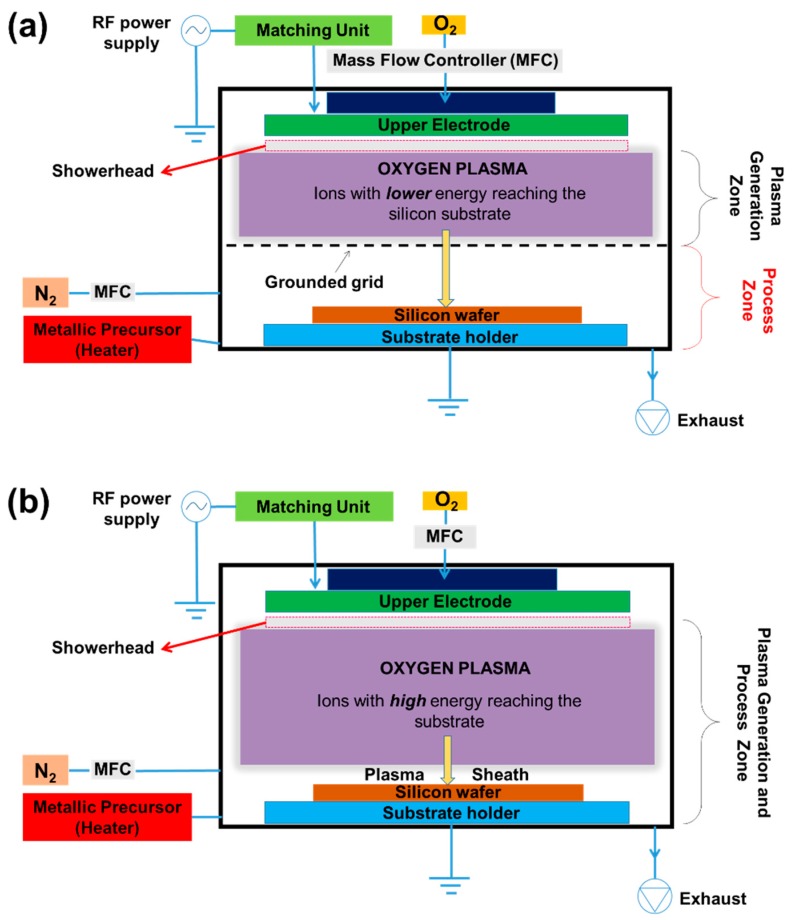
Schematic diagram of the capacitively coupled plasma reactor used for plasma-enhanced atomic layer deposition (PEALD) processes: (**a**) remote plasma mode and (**b**) direct plasma mode.

PEALD processes were performed in a Beneq TFS 200 ALD system (Beneq Oy, Espoo, Finland) equipped with a capacitively coupled plasma reactor, as illustrated in the schematic diagram shown in Figure 1. The reactor consists of a cylindrical chamber, with a 200 mm diameter, composed by two zones: (i) the upper plasma generation zone, where the plasma is generated by capacitive coupling of the upper plate with a 13.56 MHz RF power supply (Cesar, Advanced Energy Inc., Fort Collins, CO, USA) and grounding the bottom grid electrode plus radial reactor walls; and (ii) the lower process zone (sample stage), where there is no plasma generation. As the plasma generation zone is located at a distance from the process zone, the ion damage should be significantly suppressed [46]. In our experiments, the spacing between the top plate and the grid was 30 mm, whereas between the grid and the substrate holder, it was 12 mm (Figure 1a). In the second plasma exposure configuration shown in Figure 1b, we removed the grid, and the sample was exposed to the plasma generation zone. Thus, the damage caused by the ion impact on the substrate surface must be higher [46]. These PEALD reactor configurations schematized in Figure 1a,b are named remote plasma mode and direct plasma mode, respectively [38].

The substrates used for TiO_2_ thin-film deposition were three-inch p-type <100> silicon wafers (UniversityWafer Inc., South Boston, MA, USA) chemically cleaned, using a modified RCA recipe described in [47]. Between each bath, the Si wafers were washed in deionized (DI) water for 5 min. Subsequently, they were dipped in diluted hydrofluoridric acid, in the proportion 80:1 (80 H_2_O + 1 HF (49%)), at room temperature, for 100 s, and were rinsed in DI water for 3 min.

TiO_2_ thin films were grown using titanium (IV) isopropoxide (TTIP, 97.0%, Sigma-Aldrich, São Paulo, Brazil) as a metallic precursor and O_2_ gas (99.99%, White Martins, Jacareí, Brazil) to generate the O_2_ plasma (oxidant precursor). Here, the TTIP precursor was heated at 70 °C to obtain a high vapor pressure, and nitrogen (99.999%, White Martins, Jacareí, Brazil) was used as the gas of purge. The vapor delivery line of the TTIP was also heated to 70 °C, to prevent precursor condensation. The insertion of the oxygen gas was through the upper plate of the plasma generation zone at a flow rate of 50 sccm. Before the deposition process, the reactor chamber was evacuated at a pressure of 10^−2^ mbar, and the N_2_ gas purge was maintained around 1.0 mbar through the insertion of 250 sccm. The reactor temperature was fixed at 250 °C for all processes, and its variation during the film growth did not exceed 3 °C. For PEALD TiO_2_ processes, the reaction cycle number was maintained at 1000. The self-bias voltage varied by approximately 70–100 V during the 100–150 W O_2_ plasma pulse. This voltage drop value in the plasma sheath (see Figure 1b) is sufficient to supply energy to the ions, to induce chemical reactions in growing film during PEALD [48].

The experimental procedure for the deposition of TiO_2_ thin films was divided into three sets of experiments. In the first set, the RF power was varied between 100 to 150 W. Concomitantly, in the second set the plasma exposure mode was varied to study the impact of direct plasma exposition during the ligand pulse. In the third set, Al_2_O_3_ partial-monolayers were grown with TiO_2_ by alternating cycles of TiO_2_ and Al_2_O_3_ in supercycles, in order to form a TiO_2_/Al_2_O_3_ nanolaminate. For this latter, the experimental parameters are detailed in the next subsection.

### 2.2. Nanolaminate Preparation

TiO_2_/Al_2_O_3_ nanolaminate structure was prepared, using the design proposed by Testoni et al. [49], i.e., alternatively depositing a TiO_2_ sublayer and Al_2_O_3_ partial-monolayer, respectively, in “*n*” supercycles. The Al_2_O_3_ is formed by a single cycle of trimethylaluminum (TMA)–O_2_ plasma, so it is a partial-monolayer because of steric hindrance of the precursors, while the TiO_2_ sublayer is formed by repeating 60 cycles of TTIP–O_2_ plasma. This condition is in the interface of the TiO_2_ crystallization disruption [49]. Here, the Al_2_O_3_ partial-monolayers were grown, using TMA (97%, Sigma-Aldrich, São Paulo, Brazil) at 21 °C and O_2_ plasma. TiO_2_ sublayers were deposited, using TTIP at 70 °C and O_2_ plasma. High purity N_2_ was used as purge and carrier gas for both TMA and TTIP precursors. The base pressure of the reactor was below 10^−2^ mbar, and, during the deposition, the gas pressure was maintained around 1.0 mbar through the insertion of 300 sccm of nitrogen. The TiO_2_/Al_2_O_3_ films were grown under the following conditions of supercycle: 1 cycle of TMA–O_2_ plasma per 60 cycles of TTIP–O_2_ plasma. The supercycle was repeated until 2700 cycles of TTIP–O_2_ plasma, resulting in 45 layers of TiO_2_/Al_2_O_3_. The temperature and RF power were fixed at 250 °C and 100 W, respectively, and the PEALD reactor was operated in remote mode.

### 2.3. Thin-Film Characterization

Rutherford backscattering spectroscopy (RBS) was used to determine the film thickness and film elemental composition (in at. %). The measurements were performed in a Pelletron-type accelerator, using a 2.2 MeV 4He^+^ beam, and the particle detector was positioned at an angle of 170° to the incident beam. The detection sensibility of RBS in relation to Ti, O, Al and Si is approximately 5%. The RBS data were analyzed, using the computer code MultiSIMNRA [50,51]. The theoretical density considering the TiO_2_ crystal structure was applied to convert the RBS density values (10^15^ atoms.cm^−2^) into the thickness (nm) of the layer. Raman scattering measurements were used for microstructural analysis of the samples. The Raman spectra were recorded at room temperature, with a Raman microspectrometer (Horiba, Evolution, Kyoto, Japan) equipped with a thermoelectrically cooled multichannel charge-coupled device detector. The spectral resolution was better than 1 cm^−1^ over the range from 100 to 900 cm^−1^, and the power of the incident laser beam on the samples was <10 mW, with an excitation wavelength of 532 nm. Phonons modes were then analyzed by fitting Raman peaks with a Voigt profile, fixing the Gaussian linewidth (1.6 cm^−1^) to the experimental setup resolution. In order to characterize the crystalline structure, the grazing incidence X-ray diffraction (GIXRD) method was used. GIXRD patterns were obtained at room temperature, in a Shimadzu XRD 6000 goniometer, using cooper target (CuK_α_ radiation 1.5418Å), 2θ from 20° to 80°, at a scanning speed of 0.02°/s, a voltage of 40 kV and a current of 30 mA. Moreover, the GIXRD studies were carried out at an incidence angle of 0.29°. The morphology of the samples was evaluated in a Tescan Mira (TESCAN Brno, s.r.o., Kohoutovice, Czech Republic) field emission scanning electronic microscopy (FESEM), coupled with an AZtec 3.1 energy-dispersive X-ray spectrometer (EDS).

### 2.4. Fabrication and Characterization of the MOS Structures

**Figure 2:**
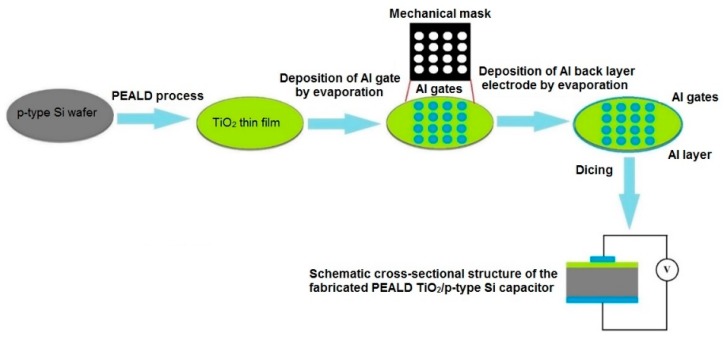
Schematic representation of the fabrication process of the PEALD TiO_2_/p-type Si capacitors.

Aluminum (Al) was evaporated on the PEALD TiO_2_ films through a mechanical mask, to form Al gates with area of 4.3 × 10^−3^ cm^2^ and thickness of approximately 200 nm. Subsequently, aluminum layers with a thickness of approximately 200 nm were evaporated on the backside of Si substrate. Thus, the MOS structure of the PEALD TiO_2_/p-Si capacitors were formed. This fabrication process is illustrated in Figure 2.

The dark current density-voltage (J-V) characteristics of the PEALD TiO_2_/p-Si capacitors were measured at room temperature by an Agilent 4146 C source measurement unit (Keysight Technologies, São Paulo, Brazil) programmed to apply potentials on the metal gate between −10 V and +10 V in steps of 0.1 V/s. The capacitance-voltage (C-V) and conductance-voltage (G-V) characteristics of the capacitors were evaluated at 1 MHz and at room temperature, using an HP 4280A C meter/C-V plotter (Hewlett-Packard Inc., Palo Alto, CA, USA). Both types of equipment mentioned above are highly accurate with a low noise ground unit.

## 3. Results and Discussion

### 3.1. Film Characterization

#### 3.1.1. Chemical Composition

**Figure 3:**
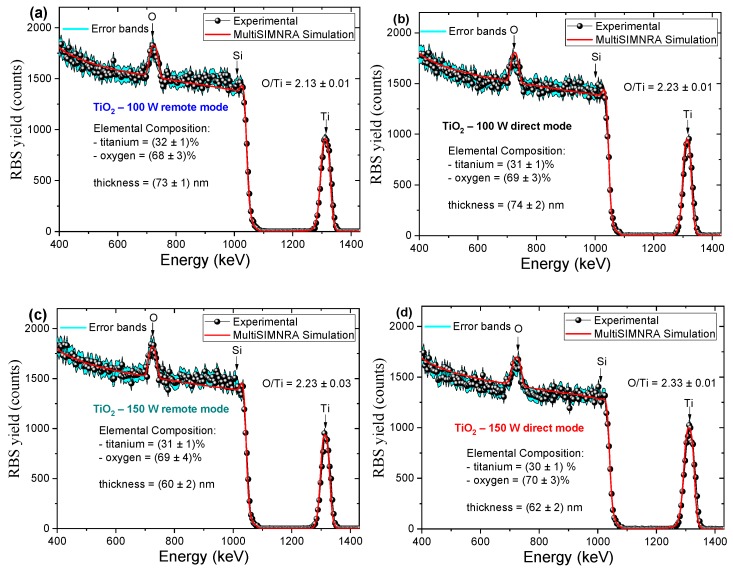

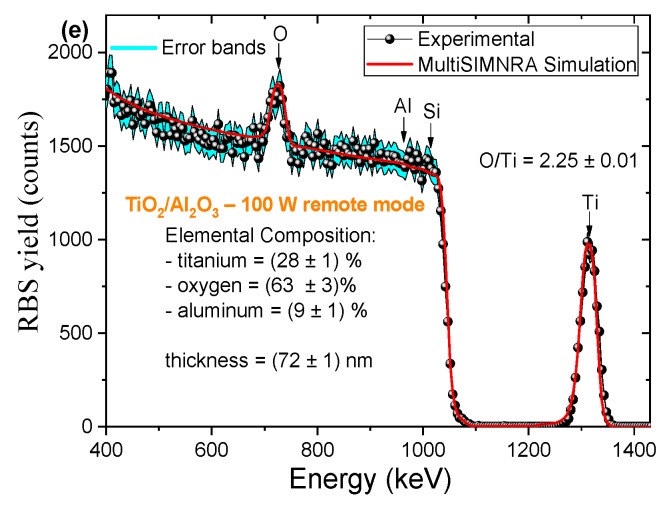
Experimental, simulated RBS spectra and data error bands for PEALD TiO_2_ films deposited under different conditions: (**a**) 100 W in remote mode, (**b**) 100 W in direct mode, (**c**) 150 W in remote mode, (**d**) 150 W in direct mode and (**e**) 100 W in remote mode (TiO_2_/Al_2_O_3_ nanolaminate). The precision was fixed in the MultiSIMNRA, at 10^−7^, with the error bars in the simulated RBS spectra being smaller than the line size.

Studies of stoichiometry throughout the TiO_2_ and TiO_2_/Al_2_O_3_ films’ thickness were done, using RBS. Figure 3 shows the experimental and simulated RBS spectra for TiO_2_ films deposited under different conditions of RF power, plasma mode and Al_2_O_3_ partial-monolayer insertion.

The backscattered signal from the TiO_2_ film on the Si substrate is characterized by well-defined peaks of Ti and O. These peaks depend on the backscattering cross-section, and the areas below the peaks represent the areal density of the atom. Most of the time, the peak position is not matched with the exact energy position of the atom. The Si substrate in the spectra is correlated with the pattern similar to a shoulder. Note that, in the case of TiO_2_/Al_2_O_3_ nanolaminate, the Al peak is not evident; however, it is necessary to consider 9% of its elemental composition during the simulation of the experimental RBS spectrum, as shown in Figure 3e. This behavior occurs because (a) Si and Al have very close atomic numbers that cause an overlapping of their respective peaks, and (b) the amount of Al in the film is low due to the nature of the nanolaminate PEALD process that used a single cycle of TMA-O_2_ plasma, while repeating 60 cycles of TTIP-O_2_ plasma during each super-cycle. This results in a low amount of Al in the film (9%), along with the reduced peak intensity in the RBS spectrum. The plasma mode, under the same RF power, does not significantly influence the film thickness. On the other hand, if the RF power is increased, the film thickness is reduced for both plasma modes. This behavior for the TTIP precursor was demonstrated in our previous study [45], where it was observed that the growth per cycle (GPC) decreases when RF power is increased. The leading cause is related to the increase of the density of O_2_ radicals that fragments the TTIP-ligands, hindering the formation of Ti-O bonds due decreasing of the free path.

The insertion of Al_2_O_3_ partial-monolayers in TiO_2_ using the nanolaminate design proposed by Testoni et al. [49] showed that a considerable increase of number of cycles (2700 cycles) was necessary for obtaining a film thickness near to pure TiO_2_ condition (1000 cycles). This is due to the poisoning effect promoted by the TMA pulse, affecting the growth kinetics of subsequent TiO_2_ layers, and thereby altering the overall growth per cycle (GPC) of TiO_2_/Al_2_O_3_ nanolaminate [49].

The elemental chemical composition of the samples is shown, together with the RBS spectra, in Figure 3. The TiO_x_ films show an excess of oxygen, i.e., x values ranging from 2.13 ± 0.01 to 2.33 ± 0.01, as can be seen in Figure 3. These results differ from those described for thermal ALD TiO_2_ films, in which either stoichometric or oxygen-deficient films are obtained [52]. The increase of oxygen content in PEALD TiO_2_ films may be related to the higher reactivity of O_2_ plasma compared to the water precursor used in thermal ALD. In a recent study, Wei et al. [5] showed the influence of temperatures on the PEALD process applied to the growth of TiO_2_ films in Si for the manufacture of MOS capacitors, where they used O_2_ plasma at 400 W of RF power. They varied the temperature from 100 to 300 °C, with steps of 50 °C. Using the X-ray photoelectron spectroscopy (XPS) technique, they verified that the TiO_2_ films had O/Ti stoichiometry ranging from 2.08 to 2.32. These results are in agreement with our O/Ti stoichiometry RBS data. Raztsch et al. [35] found a slight increase in the oxygen content in TiO_2_ films grown on Si by PEALD with O_2_ plasma at 300 W. These TiO_2_ films consist of large crystallites embedded in the amorphous layer, and, through RBS, they determined the O/Ti stoichiometry of 2.00 ± 0.04. Moreover, Bousoulas et al. verified that by increasing the oxygen content in TiO_2_ films, the size of vacancy-based filaments is reduced, resulting in the more stable operation of resistive switching memory devices [53]. This can be an interesting application for the materials and devices reported in this study, especially for conditions of 100 W RF power and with the insertion of Al_2_O_3_. A more detailed explanation of the excess oxygen in the films is given in Section 3.1.2.

#### 3.1.2. Structure and Morphology

**Figure 4:**
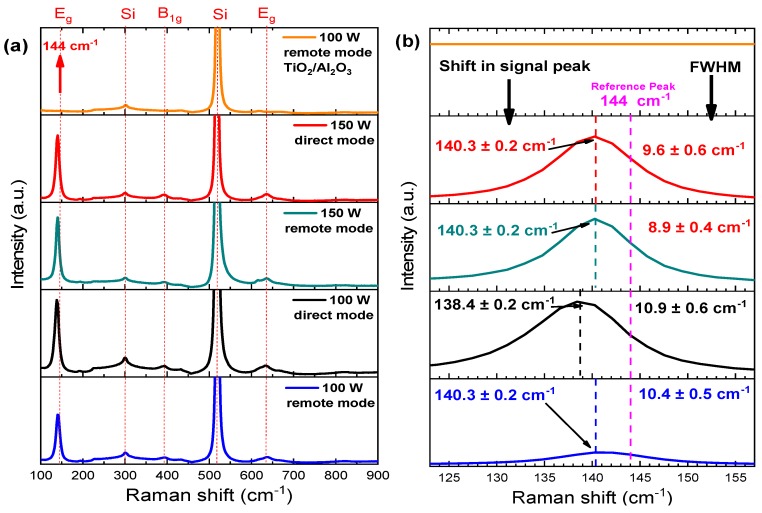
(**a**) Micro-Raman spectra for TiO_2_ films deposited under different conditions of RF power, plasma mode and Al_2_O_3_ partial-monolayer insertion and (**b**) micro-Raman spectra evidencing the shift and the FWHM of the E_g_ peak at 144 cm^−1^.

Micro-Raman spectra are shown in Figure 4a. Four Raman-active modes associated with anatase structure can be observed: A_1g_ (519 cm^−1^), B_1g_ (397 cm^−1^) and E_g_ (144 and 636 cm^−1^) with a strong peak at 144 cm^−1^ [28,54]. The crystalline condition of pure TiO_2_ thin films due to the strong peak at 144 cm^−1^ is clearly evidenced. On the other hand, for the TiO_2_/Al_2_O_3_ nanolaminate, the main peaks of the anatase phase were not observed, and the rutile phase is formed above 500 °C for silicon substrate [28,37,52,53], which suggests that the film is amorphous or partially crystalline. Figure 4b shows the micro-Raman spectra evidencing the shift and the full width at half maximum (FWHM) of the E_g_ peak at 144 cm^−1^. According to Parker et al. [55] and Bassi et al. [56], the shift of the E_g_ peak and bandwidth are related to non-stoichiometry, while the latter also influences the crystal size of anatase TiO_2_. Ratzsch et al. showed a slight excess in oxygen (O/Ti > 2) in high-density TiO_2_ with large crystallites embedded in the amorphous layer [35]. They observed the same behavior for the E_g_ peak at 144 cm^−1^, i.e., a shift and a broadband. Thus, the shift in the E_g_ peak and the bandwidth observed in Figure 4b for all PEALD TiO_2_ films can be attributed to the increase in the oxygen content, corroborating with RBS results. It is noteworthy that all characterizations of the thin films were performed in different positions of the samples. Therefore, the oxygen excess is not localized.

**Figure 5:**
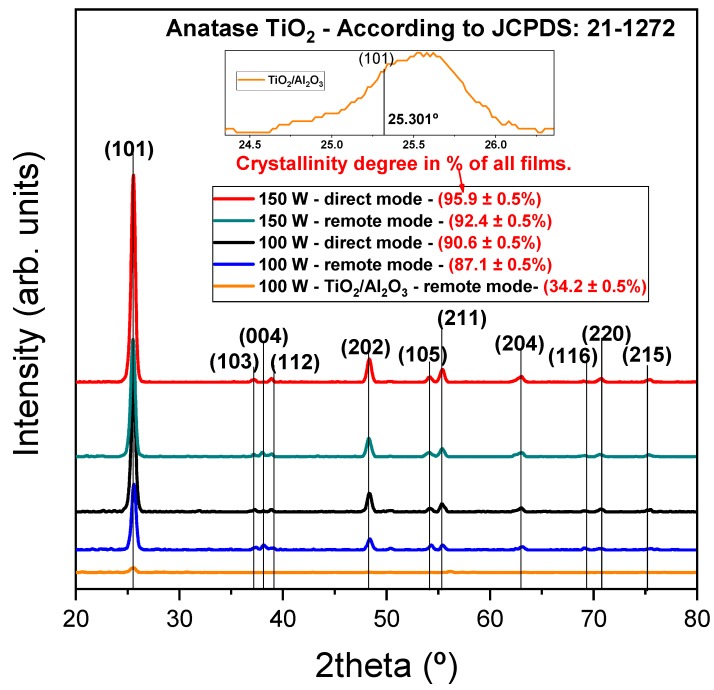
GIXRD diffractogram for TiO_2_ films deposited under different conditions of RF power, plasma mode, and Al_2_O_3_ partial-monolayer insertion. Indexed peaks are of the anatase phase. The inset figure shows the peak (101) for the 100 W—TiO_2_/Al_2_O_3_ film.

To confirm the Raman results, GIXRD measurements were performed, using the same 0.29° incident angle for all films. This angle was used to reduce the reflections from the Si substrate [45]. Based on the powder diffraction file (JCPDS: 21–1272) [57], all diffraction peaks are identified for the TiO_2_ films studied here, as shown in Figure 5.

To improve the discussion, the crystallinity degree was calculated, using the following ratio: area of all crystalline peaks/area of all peaks (crystalline and non-crystalline). As can be seen in Figure 5, the films present the following decreasing order of crystallinity degree, (95.9 ± 0.5)% to 150 W—direct mode, (92.4 ± 0.5)% to 150 W—remote mode, (90.6 ± 0.5%) to 100 W—direct mode and (87.1 ± 0.5)% to 100 W—remote mode. These results show a predominance of plasma power over the exposure mode in increasing crystallinity. On the other hand, onto the exposure mode, the direct mode has higher values compared to the remote mode. These results are probably due to the higher incidence of high energy ions for the direct mode. According to Avila et al. [58], the low intensity of the peaks indicated a lower crystallinity, when comparing the relative intensity of the peaks (101), (202) and (211), and the crystallinity degree shows the same behavior, that is, there is a decrease in intensity in the following order: 150 W—direct mode, 150 W—remote mode, 100 W—direct mode and 100 W—remote mode. To better clarify the Raman spectroscopy result on the crystallinity of TiO_2_/Al_2_O_3_ nanolaminate, the GIXRD diffractogram shows (34.2 ± 0.5)% of crystallinity degree, evidencing the partial crystallinity of the TiO_2_ layers (the inset figure shows the relative intensity of the (101) peak). Therefore, the insertion of Al_2_O_3_ partial-monolayer considerably decreases the intensity of (101) peak.

The grain size was calculated by using the Scherrer equation [59]; all the peaks of the anatase phase were considered in the calculations (Figure 5), with the values in the following order: (17.8 ± 0.7) nm to 150 W—direct mode, (19.2 ± 0.9) nm to 150 W—remote mode, (15.9 ± 0.8) nm to 100 W—direct mode and (16.2 ± 0.7) nm. These crystal sizes influence the broadband in Raman peaks [56]; however, when comparing the grain size found in the GIXRD diffractograms with the FWHM of the E_g_ peak found by the Raman spectrum, it is observed that the larger the grain size is, the smaller the FWHM is. Therefore, the film grown in 150 W—remote mode has the largest grain size with the smallest FWHM. On the other hand, the film grown with 100 W—direct mode has the largest FWHM and the smallest grain size. The results are in agreement with Parker et al. [55] and Bassi et al. [56]. Bearing in mind that there is no E_g_ peak for the TiO_2_/Al_2_O_3_ film, and the error associated with the grain size is higher than the grain value, we could not find these parameters for this film.

**Figure 6:**
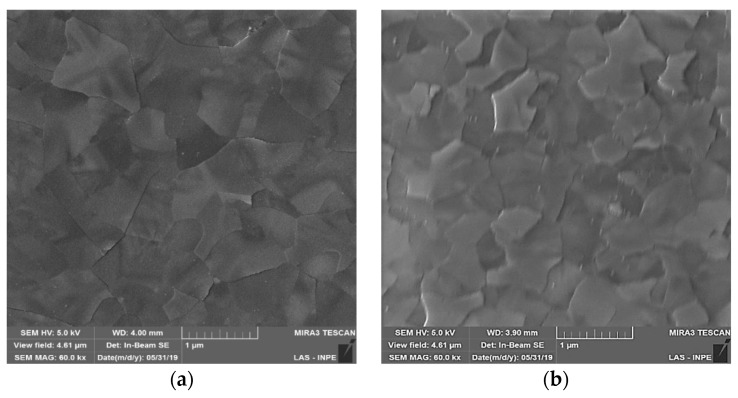

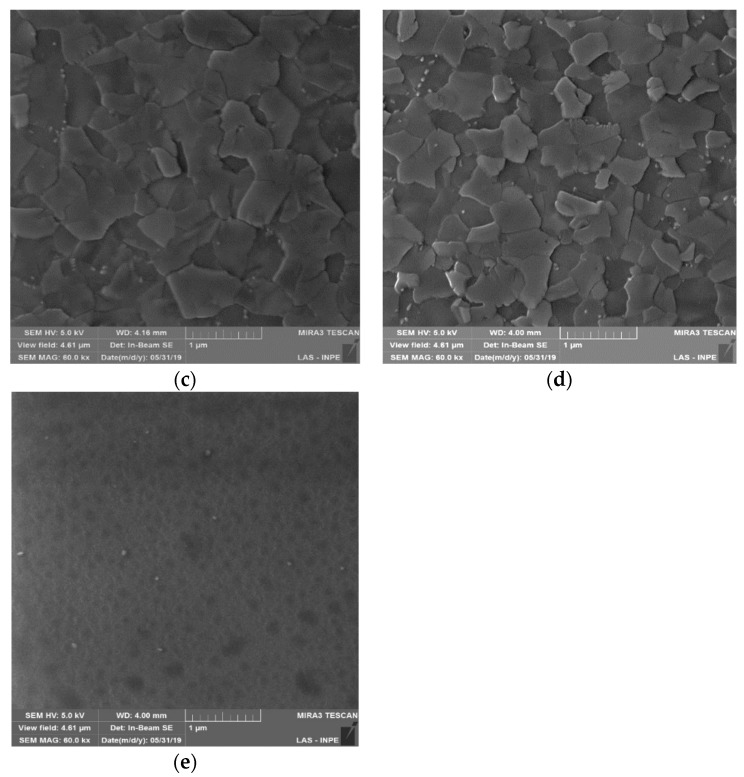
FESEM images of TiO_2_ films deposited under RF power of (**a**) 100 W in remote mode, (**b**) 100 W in direct mode, (**c**) 150 W in remote mode, (**d**) 150 W in direct mode and (**e**) 100 W in remote mode (TiO_2_/Al_2_O_3_ nanolaminate).

Figure 6 shows FESEM images of the surface of the TiO_2_ films grown at different PEALD process conditions. These images show that the grain size slight decreases with RF power for both plasma modes. The effect of the electrode grid was evidenced by the reduction of the action of the plasma ions during the capacitively coupled PEALD process [45]. As can be seen for both power values investigated, the change from remote to direct mode caused a slight reduction in grain size, probably due to the higher action of the species from plasma impinging on the substrate. An interesting result is presented in Figure 6e, where the insertion of Al_2_O_3_ partial-monolayers into TiO_2_ film disrupts the growth of crystalline grains, creating only a few nanocrystalline grains in amorphous matrix [49].

### 3.2. Electrical Characterization of TiO_2_/p-Si MOS Capacitors

#### 3.2.1. Current Density-Voltage Measurements

**Figure 7:**
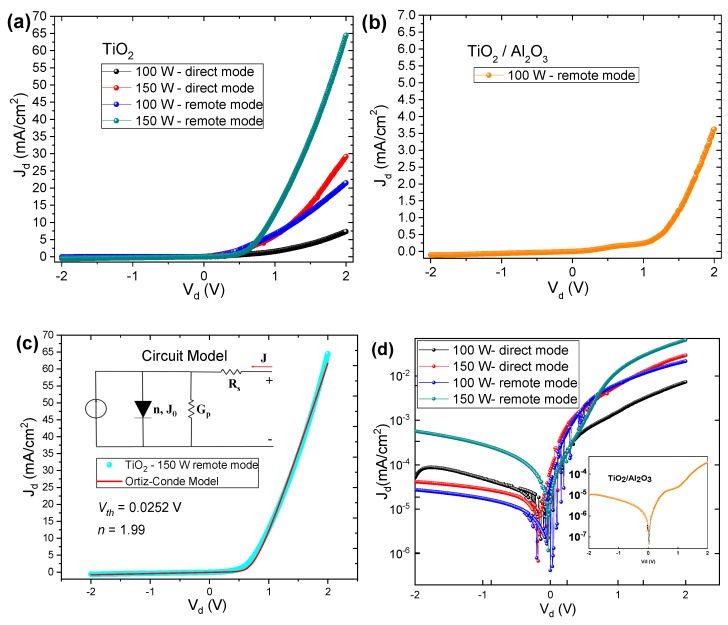
Dark J-V curves for the fabricated MOS capacitors: (**a**) TiO_2_ films grown in remote and direct mode at 100 W and 150 W, (**b**) TiO_2_/Al_2_O_3_ nanolaminate growth at 100 W, (**c**) adjustment of the curve (TiO_2_-150 W/remote mode) through Ortiz-Conde model [60] and (**d**) dark J-V curves in semi-logarithmic scale, to show the leakage current density. The value of 0.995 was used for the determination coefficient (R-squared) to fit the J-V curve, using the Ortiz-Conde model. That is, 99.5% of the dependent variable was considered, with the error bars in the simulated J-V curves being smaller than the line size.

Electrical characterization was performed on the five PEALD TiO_2_/p-type Si MOS structures fabricated at different values of RF power, substrate exposure mode and Al_2_O_3_ partial-monolayer insertion. Two sets of MOS current density were analyzed and presented in Figure 7: (i) one for TiO_2_ samples with RF powers at 100 W and 150 W for remote and direct plasma modes (Figure 7a), and (ii) another for TiO_2_/Al_2_O_3_ nanolaminate (Figure 7b). Figure 7d shows the dark J-V curve in semi-logarithmic scale under positive and negative biases at room temperature. As can be seen, the leakage current density at −2 V is in the order of 10^−5^~10^−4^ A/cm^2^, which is in the same order of magnitude reported by Wei et al. [5] and Baek et al. [1] that used PEALD and tetrakis dimethylamino titanium (TDMAT) as precursor to growth TiO_2_ (20 nm) on Si.

To study the diode ideality factor (*n*), we used the dark J-V-adapted Ortiz-Conde model [60], which considers the equivalent circuit model, consisting of a single exponential type ideal junction, a series parasitic resistance (*R_s_*) and a parallel parasitic conductance (*G_p_*), as shown in Figure 7c. The *J*(*V*) function of the equivalent circuit model is given by the following equation:

(1) $$J=J_{0}\left(\exp\left(\frac{V-JR_{s}}{nV_{th}}\right)-1\right)+\left(V-JR_{s}\right)G_{p}$$

where *J* is the current density, *V* is the terminal voltage, *J*_0_ is the reverse saturation current density and Vth=kTq is the thermal voltage.

This implicit transcendental function cannot be explicitly resolved by using a standard elementary function. The solution depends on a particular function known as the *Lambert W* function [61]. By definition, this function is defined as the solution of the *Lambert W (x) exp (Lambert W(x)) = x*, where the solutions for each variable, *J* and *V*, are an explicit function of the other. Using the auxiliary Equation (1), we obtain the parameters *a*, *b*, *c* and *d* as a function of the equivalent circuit model:

(2) $$a=\frac{1+R_{s}G_{p}}{J_{0}}$$

(3) $$b=\frac{J_{0}-V.G_{p}}{J_{0}},$$

(4) $$c=\frac{-R_{s}}{nV_{th}},$$

(5) $$d=\frac{V}{nV_{th}},$$

The general solution is as follows:

(6) $$J=\frac{nV_{th}}{R_{s}}LambertW\left(\frac{J_{0}R_{s}}{nV_{th}\left(1+R_{s}G_{p}\right)}\exp\left(\frac{V+J_{0}R_{s}}{nV_{th}\left(1+R_{s}G_{p}\right)}\right)\right)+\frac{VG_{p}-J_{0}}{\left(1+R_{s}G_{p}\right)}$$

This explicit analytic expression can be used to extract model parameters (Table 1) directly from the fit of experimental data, as shown in Figure 7c.

Table 1 presents the diode ideality factor for all dark J-V curves fitted by Equation (6). As can be seen in Table 1, the ideality factor values occur between 1 and 2. Second, in the Shockley diffusion theory, which is based on the minority carrier diffusion predicted, *n* should be equal to 1 (next to an ideal junction) [62]. Sah et al. analyzed the generation and recombination in the space charge layer and predicted that *n* ≤ 2 [63]. Faulkner and Buckingham [64] proposed a theory based in traps situated in depletion layer where the values of diode ideality factor occur between 1 and 2, which was experimentally verified by Nussbaum [65]. According to the aforementioned theories, the Ortiz-Conde model adjusted the experimental curves with good precision.

For 100 W RF power, ideality factor values decreased when the exposure mode changed from direct to remote. As the physical, chemical and morphological properties of the TiO_2_ films synthesized in direct or remote mode are close, it is believed that the variation of *n* occurred because of changes in the sample’s electrical properties, as reported in Table 1. Indeed, the mode change, as well as the RF power change, modified the *R_s_* and *G_p_* values of the films, where for 100 W RF power/direct mode, the series parasitic resistance achieved 180 Ω. On the other hand, for TiO_2_/Al_2_O_3_ nanolaminate, the ideality factor was of 1.59, which suggests that the insertion of Al_2_O_3_ partial-monolayers can be used to adjust the *n* parameter. According to Jain and Kappor [66] and Shockley [62], *n* closer to 1 is more efficient for DRAM capacitors and solar cell devices. In addition, Table 1 shows that this sample has a higher series parasitic resistance of 1100 Ω.

#### 3.2.2. Capacitance-Voltage and Conductance-Voltage Measurements

C-V and G-V measurements were also performed to evaluate the electrical characteristics of the fabricated MOS capacitors. All the samples have presented a leakage current process through the TiO_2_ layer.

**Figure 8:**
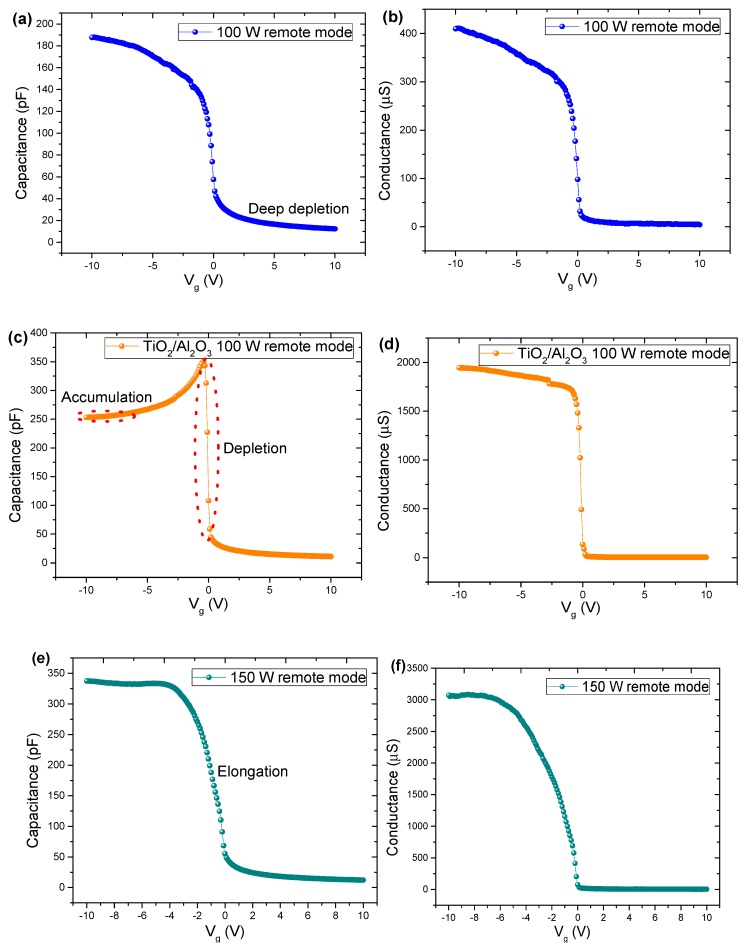
C-V and G-V curves for the fabricated MOS capacitors: (**a**) C-V curve for TiO_2_ film grown in remote mode at 100 W, (**b**) G-V curve for TiO_2_ film grown in remote mode at 100 W, (**c**) C-V curve for TiO_2_ film grown in remote mode at 100 W, (**d**) G-V curve for TiO_2_/Al_2_O_3_ film grown in remote mode at 100 W, (**e**) C-V curve for TiO_2_ film grown in remote mode at 150 W and (**f**) G-V curve for TiO_2_ film grown in remote mode at 150 W.

Figure 8 shows the C-V and G-V curves for samples deposited in remote mode. The C-V curves showed a deep depletion region for all investigated cases, indicating the absence of an inversion layer due to the high leakage current through the TiO_2_ films. By comparing the behavior of the C-V curve with the G-V curve for TiO_2_ grown at 100 W in remote mode (Figure 8a,b), it can be seen that capacitance does not stabilize on a plateau, because of increased conductance in the same region. The leakage increasing in conductance with negative voltage (Figure 8b) is related to the effect of the rise of the capacitance in this region, being an order of magnitude smaller compared to the curves in Figure 8d,f, suggesting a reduction of the series resistance in the area of accumulation [67]. The sample with Al_2_O_3_ partial-monolayer grown at 100 W (Figure 8c,d) presented a high leakage current that decreased the accumulation capacitance. This higher leakage current in the accumulation region occurs due to a negative gate bias applied to the silicon *p*-type substrate. The majority carriers are attracted to the interface Si/TiO_2_/Al_2_O_3_ with simultaneously accumulated minority carriers at the gate side flow through the TiO_2_/Al_2_O_3_ layer, thus reducing the majority carriers’ density at the interface of Si/TiO_2_/Al_2_O_3_. With the increase of gate bias, the accumulated majority carriers disappear, and a negative depletion region is formed in the silicon, which promotes a decrease of the leakage current and an increase of the capacitance, as shown by the peak in C-V curve (Figure 8c). In Figure 8e, an elongation in the C-V curve can be observed, which can be attributed to the existence of surface states in the TiO_2_ thin films [68]. This behavior may be associated with increased ion damage due to the RF power at 150 W. For TiO_2_ film growth at 150 W in remote mode, the C-V curve does not show any evidence of a decrease of the accumulation capacitance that allows to infer a drastic reduction of the leakage current. This reduction can be attributed to a structural rearrangement of the growth of the films at 150 W that reduced the series parasitic resistance (as shown in Table 1).

**Figure 9:**
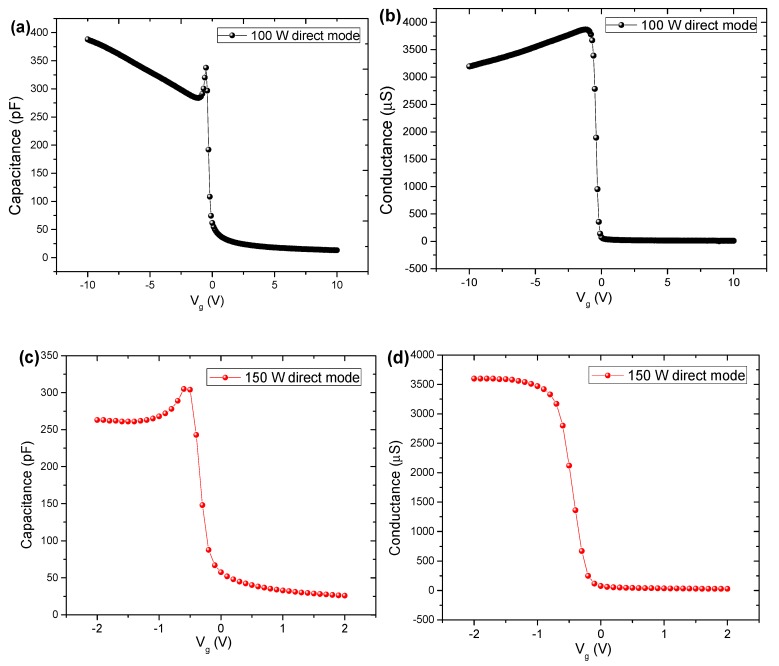
C-V and G-V curves for the fabricated MOS capacitors: (**a**) C-V curve for TiO_2_ film grown in direct mode at 100 W, (**b**) G-V curve for TiO_2_ film grown in direct mode at 100 W, (**c**) C-V curve for TiO_2_ film grown in direct mode at 150 W and (**d**) G-V curve for TiO_2_ film grown in direct mode at 150 W.

C-V and G-V curve characteristics for the samples deposited under direct exposure mode are shown in Figure 9. For capacitors with TiO_2_ films grown at 150 W in direct mode, the C-V and G-V curves were only possible to be obtained in the range of −2 V to +2 V. The C-V curves (Figure 9a,c) showed a deep depletion region for both cases, as the behaviors for PEALD TiO_2_/Si MOS capacitors with TiO_2_ films grown in direct mode indicated a current leakage through the TiO_2_ films. The sample with TiO_2_ grown at 100 W in direct mode (Figure 9a) presented a low leakage current that raised the accumulation capacitance. This low leakage current in the accumulation region occurs due to an abrupt decreasing in conductance in the same negative bias region of the gate (Figure 9b). When the gate bias increase, the accumulation of the majority carriers disappears, and a negative depletion region is formed on the silicon, promoting a decrease in leakage current and an expansion of the capacitance, as shown by the peak in the C-V curve (Figure 9a).

Compared to the 100 W TiO_2_ thin film grown in remote mode, an increase in conductance by one order of magnitude occurs, probably due to the high ion bombardment caused by the direct exposure mode (Figure 9b). The insertion of Al_2_O_3_ partial-monolayers in TiO_2_ film modulated the conductance (Figure 8d), with the maximum value of conductance at the ~2000 µS being between the values of TiO_2_-100 W in remote mode (~400 µS) and TiO_2_-100 W in direct mode (~4000 µS). It is noteworthy that for the sample grown at 150 W, in the direct mode, the same behavior in curves C-V and G-V was observed in relation to nanolaminate.

**Figure 10:**
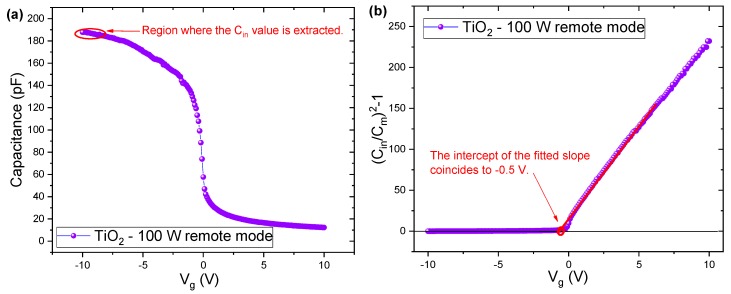
Nicollian and Brews [75] method used to calculate the capacitance of the insulator (*C_in_*), flat-band voltage (*V_fb_*) and fixed insulator charges (*Q_f_*). (**a**) C-V curve showing the extract region of the *C_in_* value, and (**b**) shows the typical V_fb_ extraction through the intercept of the fitted slope.

To better understand the electrical properties, we estimated another two parameters: (i) fixed insulator (TiO_2_ and TiO_2_/Al_2_O_3_) charges, *Q_f_*; and (ii) the interface defects density, *D_it_*, in the Si/insulator interface. We used Equation (7) to calculate *Q_f_*, and to obtain the *V_fb_*, we used the graphical method, which is shown in Figure 10 [66].

(7) $$Q_{f}=\frac{C_{in}\left(\Phi_{ms}-V_{fb}\right)}{A.q}$$

where *C_in_* is the capacitance of the insulator, *A* (4.3 × 10^−3^ cm^2^) is the front metal contact area, *q* is the elementary charge, *V_fb_* is the flat-band voltage, *Φ_ms_* is the work function difference between the work function of metal (Al) and the work function of the semiconductor (Si) that was calculated from Equation (8) [69].

(8) $$\Phi_{ms}=-0.6-\frac{kT}{q}\ln\left(\frac{N_{A}}{n_{i}}\right),$$

where *N_A_* is the doping concentration in the silicon (*N_A_* = 1.0 × 10^15^ cm^−3^), and *n_i_* is the intrinsic concentration at room temperature (*n_i_* = 1.45 × 10^10^ cm^−3^) [68,70]. To use Equation (7), it was assumed, but not yet proved, that other charges have a reduced influence on *Q_f_* measurements, and the interface traps are negligible [71,72,73,74]. The graphical method was used to find the *Q_f_* values, taking into account the average of *V_fb_* and *C_in_*. 

**Figure 11:**
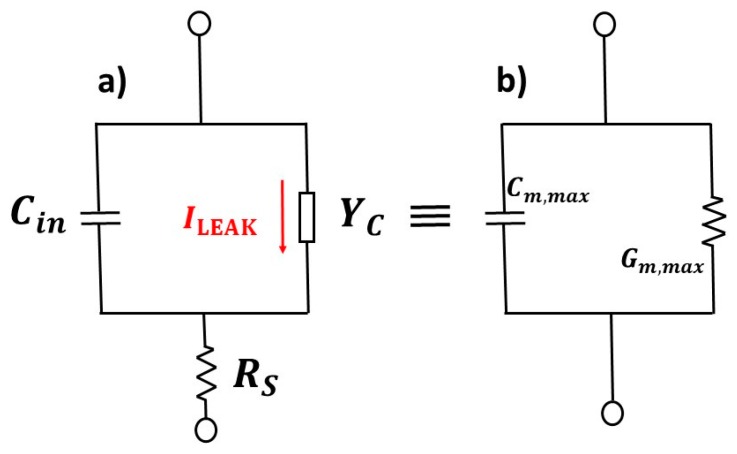
(**a**) Simplified electrical Rajab model. With *Y_C_* being the admittance, which represents the leakage process, and *R_S_* is the series resistance associated with the silicon substrate; and (**b**) measured *G_m,max_* and *C_m,max_*, which are the accumulation conductance and capacitance, respectively.

As can be seen in Figure 10a, the insulator capacitance value was extracted from the strong accumulation region in the C-V curve at negative bias due to the conductivity nature p-type of the silicon. To construct Figure 10b and extract the flat-band voltage, the following equation was used:

(9) $${\left(\frac{C_{in}}{C_{m}}\right)}^{2}-1=0,$$

where *C_m_* is the experimentally measured capacitance. To calculate *C_in_* and, consequently, *Q_f_*, in the strong leakage processes from the capacitance lowering at the accumulation region of the C-V curves (Figure 7c and Figure 8a,c), the Rajab Model was used [67]. To observe the leakage process, the Rajab model proposed a simplified electrical model, as shown in Figure 11a, where *Y_C_* is an admittance which represents the leakage process, and *R_S_* is the series resistance associated with the silicon substrate. *G_m,max_* and *C_m,max_* in Figure 11b are the accumulation conductance and capacitance, respectively, shown from a C-V meter.

Using the impedance equality of the electrical circuits shown in Figure 10a,b, we derived the equations for the leakage admittance *Y_C_* and *C_in_*, which are shown below [67]:

(10) $$Y_{C}=\sqrt{\omega C_{in}\left(-\omega C_{in}+\omega C_{m,\;max}\left(1+\frac{G_{m,\;max}{}^{2}}{{\left(\omega C_{m,\;max}\right)}^{2}}\right)\right)},$$

and

(11) $$C_{in}=\;\frac{C_{m,\;max}\left({\left(\omega C_{m,\;max}\right)}^{2}+G_{m,\;max}{}^{2}\right)}{{\left(\omega C_{m,\;max}\right)}^{2}+\left(G_{m,\;max}-R_{S}\left({\left(\omega C_{m,\;max}\right)}^{2}+G_{m,\;max}{}^{2}\right)\right)}.$$

Table 2 shows the *V_fb_* and *Q_f_* values of all samples with the front contact area of 4.3 × 10^−3^ cm^2^. As shown in the literature, TiO_2_ grown on Si generates negative *Q_f_* being more appropriate for rear passivation in solar cells, basically due to field-effect passivation [45]. Recently, Liao et al. [73] showed that TiO_x_ thin-film (63 nm) growth by ALD is also capable of providing passivation of c-Si substrates at the same level of thermal silicon oxide (SiO_2_), silicon nitride (SiN_x_) and aluminum oxide (Al_2_O_3_). This negative polarity corroborates with the best passivation performance in solar cells because negative *Q_f_* repels minority carriers (electrons), resulting in an increased level of field-effect passivation [74].

As can also be seen in Table 2, the exposure mode influences the values of the fixed charges but did not change the polarity. The increase in leakage in conductance with the negative voltage (Figure 7b) is related to the effect of the rise of the capacitance in this region of conductance that causes a reduced value in *Q_f_* around 1 × 10^11^ cm^−2^ for the TiO_2_-100 W in remote mode. The higher values were of the samples that grown in direct mode around 1 × 10^12^ cm^−2^ for both RF powers values. For the samples that grew at 100 W, a behavior similar to that of J-V curves was observed where the Al_2_O_3_ partial-monolayer modulated the values of the ideality factor. In this case, the Al_2_O_3_ partial-monolayer obtained a magnitude above the *Q_f_* value obtained for TiO_2_-100 W in remote mode and one order of magnitude below the value obtained for the TiO_2_-100 W in direct mode (as shown in Table 2), acting as a modulator between the two cases. As can be seen in Table 2, this analysis indicates that the fixed insulator charge values are not intrinsic to each insulator, and its concentration can be modified by exposure mode and plasma power.

Another critical parameter for the interface is the density of interface defects (*D_it_*). For this, it was used the Hill–Coleman model [74], a single-frequency approximation for interface defects density determination, as shown below:

(12) $$D_{it}=\frac{2}{A.q}\frac{\frac{G_{m,\;max}}{\omega }}{{\left(\frac{G_{m,max}}{\omega .C_{in}}\right)}^{2}+{\left(1-\frac{C_{m,max}}{C_{in}}\right)}^{2}},$$

where *G_m,max_* is the maximum experimentally conductance, *C_m,max_* is the maximum experimentally capacitance and *ω* = 2π*f* (*f* = 1 MHz).

The advantage of this model is that only three measured values (*G_m,max_*, *C_m,max_* and *C_in_*) and a single frequency are needed. Therefore, the approximation is realized from the need of C-V and G-V plots. Table 3 summarizes the *D_it_* values extracted with the help of Equation (12).

Most of the samples show *D_it_* values within the same order of magnitude, being the exception of the TiO_2_-100 W in remote mode. This exception probably occurs due to the approximation technique for *D_it_* is within 25–30% of the Nicollian–Brews peak *D_it_*’s value between the flat band and mid-gap [71]. Since *D_it_* is known to change by order of magnitude or more in the region between the flat band and mid-gap, this approximation is reasonable [74]. As suggested by Hill–Coleman, the frequency is into the MHz region. The results listed in Table 2 and Table 3 show that the samples exhibit a density of interface defects (~10^11^–10^13^ cm^−2^) and a high density of negative fixed charges (~10^11^ eV^−1^.cm^−2^), which make them potential candidates for application in solar cells and DRAM technology. For example, TiO_2_-100 W and TiO_2_-150 W growth by the direct mode presents higher *Q_f_* and lower *D_it_*. These combined characteristics show the quality of these films to act as a passivation layer in solar cells. Cunha et al. [42] and Kotipali et al. [72] showed values in the same order of magnitude for *Q_f_* (~10^11^–10^12^ cm^−2^) and *D_it_* (~10^10^–10^12^ eV^−1^ cm^−2^) for other thin films, namely Al_2_O_3_, Si_3_N_x_ and SiO_x_. In both works, the films were grown on Si and CIGS.

To improve the discussion, we compared our results with works that used chemical and thermal treatments to enhance the electrical properties of the following structures Al_2_O_3_/Si and TiO_2_/Al_2_O_3_/Si. Yoshitsugu et al. [76] deposited Al_2_O_3_ (25.2 nm) on Si by PEALD at a fixed temperature of the 100 °C and RF power at 400 W. They used high-pressure deuterium oxide annealing (HPDOA) treatment and reduced the leakage current density to an order of 10^−7^ A/cm^2^. This result is two orders of magnitude lower than our results. Zougar et al. [77] deposited Al_2_O_3_ (~1 nm) on Si by the ultrasonic spray method. They used post-deposition annealing and post-metallization annealing as surface treatment. They showed values of for *Q_f_* (~10^10^–10^11^ cm^−2^) and *D_it_* (~10^11^–10^12^ eV^−1^ cm^−2^). Both results show a lower quality of these films in comparison with our films. Baek et al. [1] grew Al_2_O_3_ (50 nm), TiO_2_ (50 nm) and Al_2_O_3_/TiO_2_ (50 nm) on Si by PEALD at 100 °C and with RF power fixed at 200 W. They used several chemical treatments to modify the surface. The leakage current density values of Al_2_O_3_ and Al_2_O_3_/TiO_2_ are, respectively, 10^−8^ and 10^−9^ A/cm^2^. These values are lower than our results, but they showed a leakage current density of the TiO_2_ of the order of magnitude of 10^−3^ A/cm^2^, which is two orders of magnitude higher than our values. Even without thermal or chemical treatment, our films have a high electrical quality, as shown in this brief comparison.

## 4. Conclusions

TiO_2_ thin films were deposited on p-type Si substrates under different conditions by PEALD technique. Structural, chemical and morphological properties of the as-grown films were studied as a function of the following deposition parameters: RF power, substrate exposure mode and Al_2_O_3_ partial-monolayer insertion. Chemical composition determined by RBS analysis showed that the TiO_x_ films have an excess of oxygen content, with x values ranging from 2.13 ± 0.01 to 2.33 ± 0.01, which can be related to the higher reactivity of O_2_ plasma. It was also observed that, with the insertion of Al_2_O_3_ partial-monolayers in TiO_2_ to form the nanolaminate structure, an increase in the number of cycles to 2700 is necessary to obtain a film thickness close to pure TiO_2_. GIXRD diffractograms showed a crystalline structure for all pure TiO_2_ thin films with a crystalline degree of 87.1% to 95.9%. The Al_2_O_3_ partial-monolayer reduced TiO_2_ crystallinity degree to 34.2%. Raman spectra allow us to observe that the shift in the signal peak at 144 cm^−1^ and its bandwidth is related to the increase of oxygen content in PEALD TiO_2_ films. FESEM study evidenced the effect of the electrode grid on the reduction of the action of the plasma ions during the capacitively coupled PEALD process. It can be seen, for both power values investigated, that the change from remote to direct mode caused a slight reduction in grain size, probably due to the more significant action of species impinging from plasma to substrate. The insertion of Al_2_O_3_ partial-monolayers into TiO_2_ film disrupts the growth of crystalline grains, creating only a few nanocrystalline grains in the amorphous matrix. PEALD TiO_2_/p-type Si MOS capacitors were fabricated by depositing of Al electrical contacts. J-V, C-V and G-V measurements of these capacitors were performed. The Ortiz-Conde model was used to fit the experimental dark J-V curves and values between 1.59 to 1.99 were obtained for the ideality factor. For TiO_2_ thin-film growth at RF power of 150 W, the values did not change with the exposure mode, and this behavior suggests that the power effect is predominant in the process. For the sample TiO_2_-100 W, different values of the ideality factor were found: 1.93 (direct mode) and 1.79 (remote mode). For the TiO_2_/Al_2_O_3_ nanolaminate, the ideality factor was 1.59, suggesting that the insertion of Al_2_O_3_ partial-monolayers can tune the ideality factor. C-V and G-V curves showed a deep depletion region for all cases, which indicates the absence of a layer of inversion because of the high leakage of electrons through the TiO_2_ films. It was noted that Al_2_O_3_ partial-monolayer, grown at 100 W, modulated the conductance with the maximum value of ~2000 µS, whereas a value of ~400 µS and ~4000 µS were found for TiO_2_-100 W in remote mode and TiO_2_-100 W in direct mode, respectively. The fixed insulator charges (*Q_f_*) and the interface defects density (*D_it_*) were also estimated. In summary, the fabricated PEALD TiO_2_/p-type Si MOS capacitors exhibit electrical characteristics suitable for microelectronics and photovoltaics applications.

## Figures and Tables

**Table 1:** Series parasitic resistance (*R_s_*), parallel parasitic conductance (*G_p_*) and the ideality factor calculated by the adjusting the J-V curves by the Ortiz-Conde model.

Sample	*R_s_* (Ω)	*G_p_* (µS)	*n* (Ideality Factor)
TiO_2_-100 W remote mode	70	36	1.79
TiO_2_/Al_2_O_3_-100 W remote mode	1110	6.8	1.59
TiO_2_-100 W direct mode	180	256	1.93
TiO_2_-150 W direct mode	47	89	1.99
TiO_2_-150 W remote mode	50	84	1.99

**Table 2:** *V_fb_* and *Q_f_* calculated by using Nicollian and Brews method and the Rajab model.

Sample	*V_fb_* (V)	*Q_f_* (cm^−2^)
TiO_2_-100 W remote mode	−0.5	−3.54 × 10^11^
**TiO_2_/Al_2_O_3_-100 W remote mode**	−0.4	−8.41 × 10^11^
TiO_2_-100 W direct mode	−0.2	−2.41 × 10^12^
TiO_2_-150 W direct mode	−0.4	−2.10 × 10^12^
TiO_2_-150 W remote mode	−0.3	−8.80 × 10^11^

**Table 3:** Density of interface defects (*D_it_*) calculated by Hill–Coleman model.

Sample	*D_it_* (eV^−1^.cm^−2^)
**TiO_2_/Al_2_O_3_-100 W remote mode**	5.52 × 10^11^
TiO_2_-100 W direct mode	7.08 × 10^11^
TiO_2_-100 W remote mode	2.44 × 10^11^
TiO_2_-150 W direct mode	3.48 × 10^11^
TiO_2_-150 W remote mode	6.74 × 10^11^
